# Adherence to iron and folic acid supplementation and prevalence of anemia among pregnant women attending antenatal care clinic at Tikur Anbessa Specialized Hospital, Ethiopia

**DOI:** 10.1371/journal.pone.0232625

**Published:** 2020-05-04

**Authors:** Beshir Bedru Nasir, Atalay Mulu Fentie, Mistr Kindu Adisu

**Affiliations:** Department of Pharmacology and Clinical Pharmacy, School of Pharmacy, College of Health Sciences, Addis Ababa University (AAU), Addis Ababa, Ethiopia; University of Cambridge, UNITED KINGDOM

## Abstract

**Introduction:**

Anemia during pregnancy has a significant adverse effect on both the mother and fetus. Iron and folic acid supplementation (IFAS) is the feasible and cost effective strategy to control and prevent anemia in pregnancy. However, the success of this strategy is suboptimal due to poor maternal adherences to the regimen. The aim of this study was to assess prevalence of anemia, rate of adherence to IFAS and associated factors among pregnant women at Tikur Anbessa Specialized Hospital (TASH), Ethiopia.

**Method:**

Institution based cross sectional study was conducted among 250 pregnant women who were selected using systematic random sampling from antenatal care clinic (ANC) attendants of TASH. Data was collected through interview and medical chart review by using structured questionnaire. The data was analyzed by SPSS v.24. Binary logistic regression was used to identify the associated factors for IFAS and *P < 0*.*05* was used to declare the association.

**Results:**

The prevalence of anemia was 4.8% and half of the study participants were knowledgeable about anemia. The rate of adherence to IFAS was 63.6%. Forgetfulness and fear of side effect were the commonest reasons for poor adherence to IFAS. Gestational age at first ANC visit and educational level were significantly associated with adherence to IFAS. Thus, pregnant women who started their ANC follow up at first trimester (AOR = 1.87, 95% CI (1.18–3.36)) and education level of college and above (AOR = 4.236, 95% CI (1.35–13.25)) and completed secondary education (AOR = 4.09, 95% CI (1.39–12.02)) were more likely to be adherent to IFAS compared with their comparators.

**Conclusion:**

Even though anemia prevalence was very low among the study participants, adherence to IFAS was still a challenge during pregnancy. Therefore, counseling about IFAS and anemia prevention and promoting the benefits of early ANC visit are recommended to improve adherence to IFAS.

## Introduction

Anemia is a hematologic disorder characterized by a decline in the concentration of circulating erythrocytes or hemoglobin in the blood [1]. Among pregnant women anemia is defined as hemoglobin (Hb) concentration of less than 11 g/dl [2]. It is a global public health problem especially in the developing countries with diverse impact across different population groups typically pregnant women [3]. Iron and folate deficiency are the major causes of nutritional anemia [4]. During pregnancy iron and folate requirements is high and the amount of iron absorbed from the diet is not sufficient to meet the their requirements [5]. Therefore, pregnant women are at high risk of developing anemia due to iron and folate deficiency if there is not maternal supplementation during the pregnancy [6].

World Health Organization (WHO) reported that, 38.2% of global and 46.3% of African region pregnant women are affected by anemia [7]. In Ethiopia, the prevalence is varied ranging from 23% to 31.7% [1, 8], which seems lower than the WHO reports. However, there are reports that depicts Ethiopia is one of the countries in which highest maternal and child mortalities are documented possibly due to poor maternal services utilization like micronutrient supplementation [9].

Anemia is associated with different negative consequences to the mother and fetus such as fatigue, impaired immune function, increased risk of cardiac diseases due to insufficient Hb and death in cases of hemorrhage during labor. In addition, it can cause miscarriages, stillbirths, prematurity and low birth weight resulting from insufficient supply oxygen, to the fetus [10–12]. Furthermore, it is associated with increased risk of neural tube defect, preeclampsia, fetal malformations and preterm delivery [13]. Overall, anemia contributes to 18% of perinatal mortality, 19% of preterm births, and 12% of low birth weight in developing countries like Ethiopia [14].

Iron and folic acid supplementation (IFAS) with optimal adherence is the main cost-effective strategy for prevention and control of iron and folate deficiency anemia during pregnancy [15]. IFAS during pregnancy was shown to reduce the risk of all types of maternal anemia by 70% and iron deficiency anemia by 57% [16]. WHO recommends all pregnant women should receive and consume a standard dose of 30-60mg iron + 400 μg folic acid daily supplement starting as early as possible and should be taken throughout pregnancy to control and prevent micronutrient deficiencies [16]. Accordingly, many developing countries including Ethiopia implemented this strategy through antenatal care (ANC) programs. However, only few countries have reported significant improvement in IFAS and anemia control and prevention. There are several factors, that includes poor knowledge and awareness about anemia during pregnancy, inaccessibility of IFAS and fear of side effects, believed to be responsible for not conforming to the recommended IFAS during pregnancy [17]. Several studies in different regions of Ethiopia revealed the existence of poor adherence to IFAS during pregnancy [18–20]. In addition, Ethiopian demographic health survey of 2016 reported that only 5% pregnant women took an iron with a folic acid tablet for 90 days and 58% of pregnant women did not at all during their time of pregnancy [21]. Low utilization of ANC services, inadequate supply of IFA tablets, poor counseling and lack of knowledge on anemia were the identified associated factors for poor adherence to IFAS [22, 23]. The presence of low level of adherence alarms the need to study the factors influencing adherence to IFAS to improve outcome of the current strategy [20]. Furthermore, majority of the previous findings in Ethiopia regarding adherence to IFAS and prevalence of anemia were from rural areas with relatively lower access to health care that might not reflect the real scenario in the capital cities. Therefore the aim of this study was to determine prevalence of anemia, rate of adherence to IFAS and its associated factors that might be an important step to improve IFAS use among pregnant women and prevent related complications.

## Methods and materials

### Study setting and study design

The study was conducted at ANC clinic of Tikur Anbessa Specialized Hospital (TASH), which is the largest teaching referral hospital located in the capital city of Ethiopia. TASH serves as training center for undergraduate and postgraduate medical students and provides different clinical services in its specialty clinics. ANC, a division of the department of gynecology/obstetrics, serves for more than 1025 clients per month. An institution based cross-sectional study was conducted from 05 April to 05 June, 2019.

### Study population and sampling procedures

The source populations were all pregnant women attending ANC service at TASH during the study period. All pregnant women who had at least two visits of ANC for the current pregnancy, previously supplemented with 60mg iron with 0.4mg folic acid tablets for at least one month before the date of interview and willing to participate in the study were included. Pregnant women with mental disorder, unable to hear and/or speak, and very sick were excluded from the study.

The sample size was calculated by using single population proportion formula by considering the expected prevalence of adherence to IFAS among pregnant women was 20.4% from a study done in Mecha district [19], 95% of confidence level, and 5% of marginal error. Finally a total of 257 study participants were included in the study after adding 5% of none response rate. Systematic random sampling was employed to select the study participants. First, we estimated the number of ANC service users for the previous two consecutive months (by considering two months as study period) which was 1025 mothers. Then, we calculated the K^th^ interval by dividing the estimated number of mothers attending the clinic during the study period by the calculated sample size which was 4. Finally, we interviewed the study participants at every four intervals among ANC service users and then their medical charts were reviewed.

### Data collection procedures

The data was collected using interviewer administered semi-structured questionnaire and medical charts were reviewed to check the presence of anemia. The data was collected by three pharmacists under the supervision of a senior clinical pharmacist. One day training was given to the data collectors regarding the objectives of the study and how to interview the study participants. The questionnaire was developed based on previous studies.

It was first developed in English and then translated into Amharic then translated back into English by a different person to check its consistency. The questionnaire was pretested on 5% of the sample size before conducting the actual study and appropriate correction was taken accordingly.

### Study variables and data analysis

While the dependent variable was adherence to IFAS, the independent variables were socio- demographic factors like age, religion, marital status, education level, gestational age at first ANC visit, parity, trimester, current anemia status, knowledge about anemia and IFAS.

### Data processing and analysis

The data was coded, entered and analyzed by using Statistical Package for Social Sciences (SPSS) version 24 software. Descriptive statistics such as a frequency distribution and percentages were performed to describe the demographic, socioeconomic, and obstetric characteristics of the participants. Multivariable binary logistic regression analysis was used to assess the association of the independent variables with adherence to IFAS after univariable analysis (*p*<0.2) to control confounders and *p* value < 0.05 was considered as statistically significant.

#### Ethical approval

The study was approved by Institutional Review Board of Addis Ababa University, School of Pharmacy. Verbal consent was obtained from each patient to participate in the interview and written informed consent was secured to extract data from their medical charts. Privacy and confidentiality were ensured during patient interview and medical chart review.

### Operational definitions

#### Adherent to IFAS

Pregnant mothers who took at least 65% of the expected dose of the iron-folate tablets in the previous week before the study, which is equivalent to consuming at least 1 tablet daily for 4 days in the week consecutively or consuming 20 tablets of the prescribed doses daily in a month [16, 24].

#### Anemia

A condition where the hemoglobin level in the body is less than 11 g/dl [2]

#### Good knowledge on anemia

Pregnant women were said to have good knowledge on anemia if they respond for ≥ 4 answers correctly out of seven questioned prepared to assess their knowledge of anemia.

#### Good knowledge on IFAS

Pregnant women were said to have good knowledge on IFAS if they respond at least three questions correctly out of five questions prepared to assess their knowledge of IFAS

## Results

### Socio-demographic characteristics of the participants

Among 257 participants enrolled in the study, 250 were eligible for the analysis making the response rate of 97.2% and 7 participants were excluded due to the incompleteness of the data. The mean age was 27.85 ± 5.1 years and almost all (99.2%) of the participants were married. Regarding the participants’ religion, majorities were orthodox Christian 142(56.8%) and 77(30.8%) Muslim. About 108(43.2%) of the participants had primary level education and 144(57.6%) of them were housewife (Table 1).

**Table 1:** Socio-demographic characteristics of pregnant women attending ANC follow up at TASH, Addis Ababa, Ethiopia June 2019.

Variables	Category	Frequency	Percent
**Age**	<20	17	6.8
	20–30	176	70.4
	>30	57	22.8
**Marital status**	Married	247	99.2
	Unmarried	1	0.4
	Divorced	1	0.4
**Religion**	Orthodox Christian	142	56.8
	Muslim	77	30.8
	Protestant	26	10.4
	Catholic	2	8
**Educational level**	Cannot read and write	18	7.2
	Read and write	4	1.6
	Primary education	108	43.2
	Secondary education	72	28.8
	College and above	48	19.2
**Occupation**	Housewife	144	57.6
	Government employed	45	18
	Self-employed	55	22

### Obstetrics and health-related characteristics of the participants

Majority (79.2%) of the participants were in their third trimester during the data collection and only (56%) of the respondents started ANC visit in the first trimester. About (37.2%) of the participants were primiparous and (38.8%) were multiparous. From the total samples, only 145 had complete blood count (CBC) result on their medical chart which is done for the current ANC follow up. Among them only 4.8% of the participant were anemic (Hb<11 g/dl) (Table 2).

**Table 2:** Obstetrics and health-related characteristics of pregnant women attending ANC follow up at TASH, Addis Ababa Ethiopia June 2019.

Variable	Category	Frequency	Percent
**Current gestational age**	First Trimester	9	3.6
	Second Trimester	43	17.2
	Third Trimester	198	79.2
**Gestational age when ANC was started**	First Trimester	140	56.0
	Second Trimester	99	39.6
	Third Trimester	11	4.4
**Parity**	Nulliparous	60	24.0
	Primiparous	93	37.2
	Multiparous	97	38.8
**Current hemoglobin level (n = 145)**	< 11 g/dl	7	4.8
	11–12 g/dl	19	13.1
	>12 g/dl	119	82.1

### Knowledge of the participants about anemia and IFAS

**Fig 1:**
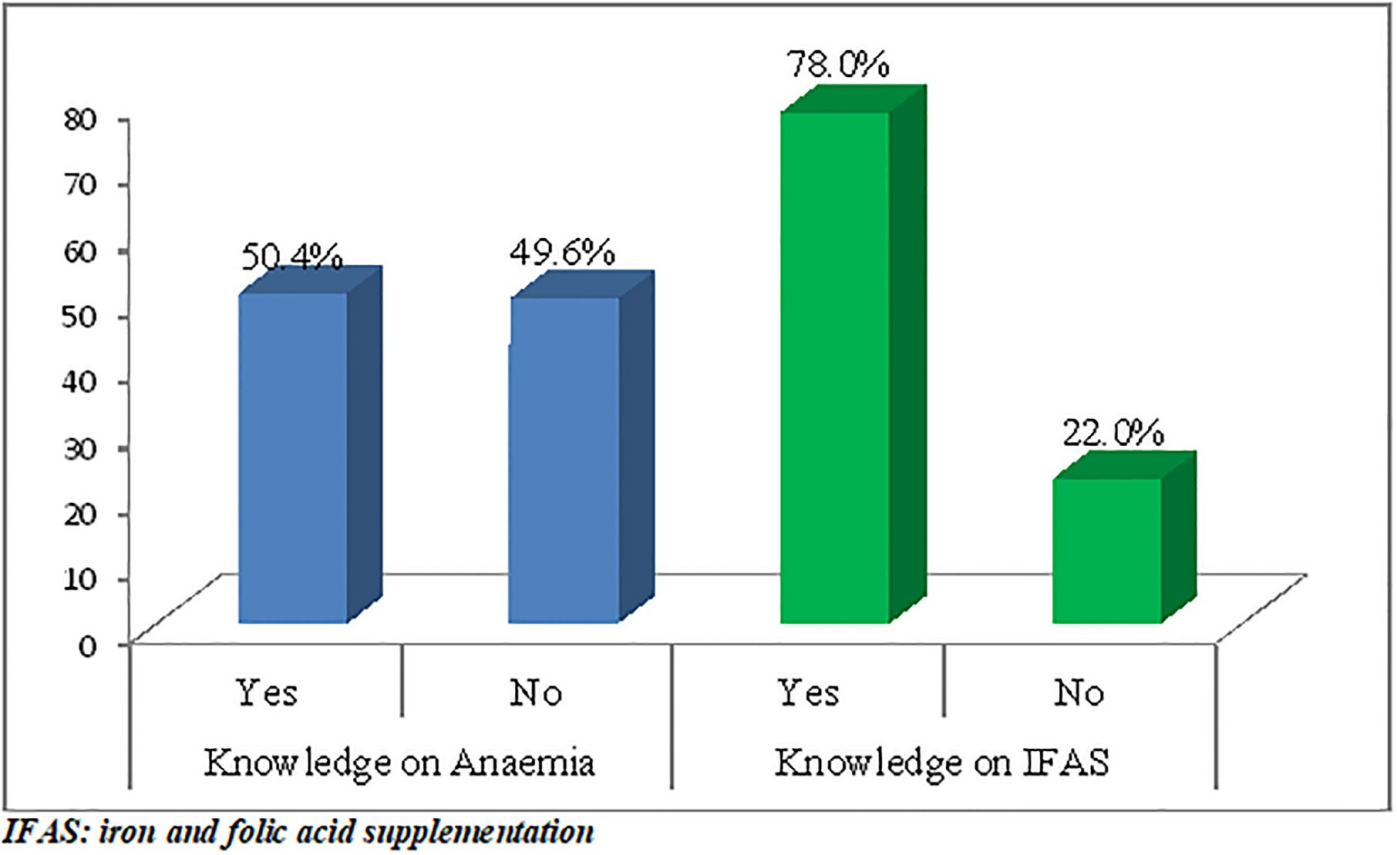
Knowledge on anemia and IFAS among pregnant women attending ANC follow up at TASH, Addis Ababa Ethiopia June 2019.

Of the total participants, 219(87.6%) had ever heard of anemia during pregnancy and only half 126(50.4%) of the respondents were knowledgeable about anemia. About 195 (78.2%) of the respondents had adequate knowledge on IFAS (Fig 1).

### Adherence to IFAS and reasons for non-adherence

**Fig 2:**
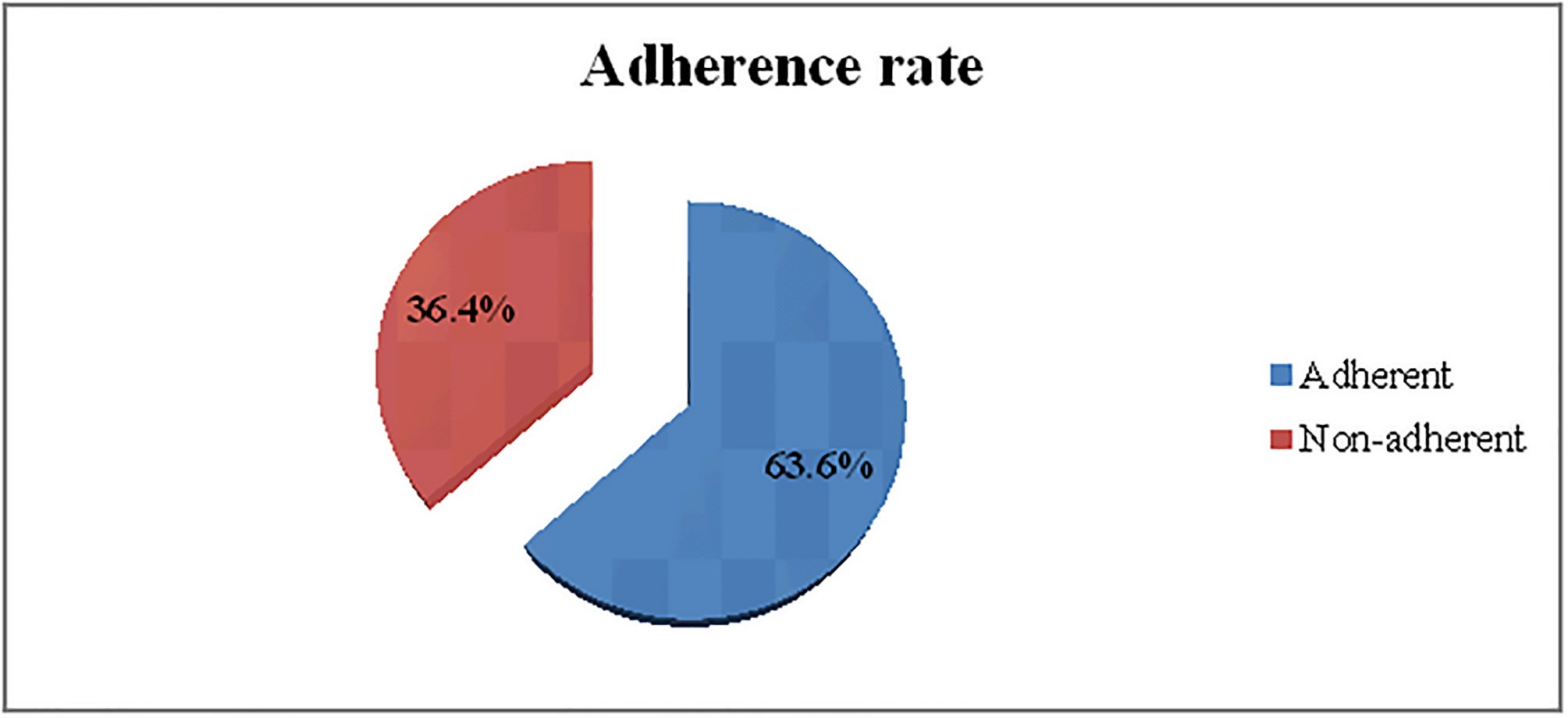
Adherence to IFAS among pregnant women attending ANC follow up at TASH, Addis Ababa Ethiopia June 2019.

The rate of adherence to IFAS among the participants was 63.6% (Fig 2).

Majority 232(92.8%) of the participants responded that advice of the health service providers was reason for taking the IFAS. Forgetfulness 76(30.4%) and fear of side effects 60(24.0%) were the major reasons for not taking the supplement (Table 3).

**Table 3:** Reasons for taking and not taking the supplement among pregnant women attending ANC follow up at TASH, Addis Ababa Ethiopia, June 2019.

	Variables	Frequency	Percent
Reason for taking IFAS	Advice of health worker	232	92.8
	Knowing it to prevent anemia	49	19.6
	Getting the tablet free	1	0.4
Reason for not taking IFAS	Fear of side effects	60	24.0
	Forgetfulness	76	30.4
	Too many pills	8	3.2
	Unpleasant tests	7	2.8
	Fear that babies will become bigger	4	1.6

### Factors associated with adherence to IFAS

From multivariable binary logistic regression analysis, only gestational age at first ANC visit and educational level showed a statistically significant association with adherence to IFAS. Pregnant women who started their ANC follow up at first trimester were more adherent to IFAS by 1.874 times as compared to first ANC visit at second or third trimester (AOR = 1.874, 95% CI (1.178–3.360)). Pregnant women with education level of college and above and secondary education were 4.236 and 4.086 times more likely to be adherent to IFAS respectively as compared to those who cannot read and write (AOR = 4.236, 95% CI (1.351–13.250) and (AOR = 4.086, 95% CI (1.389–12.017)) (Table 4).

**Table 4:** Factors associated with adherence to IFAS among pregnant women attending ANC follow up at TASH, Addis Ababa Ethiopia, June 2019.

Variables		Adherence for IFAS		COR (CI 95%)	AOR (CI 95%)
Educational level		Adherent	Non-adherent		
	Cannot read and write	7	11	1	1
	Read and write	2	2	1.97(0.28–11.06)	1.571(0.178–13.86)
	Primary education	45	63	2.93(0.43–7.13)	2.20(0.792–6.113)
	Secondary education	52	20	4.88(1.47–13.01)	4.086(1.389–12.017)*
	College and above	35	13	4.38(1.95–15.55)	4.236(1.351–13.250)*
Gestational age when ANC was started	Second or third Trimester	61	59	1	1
	First Trimester	98	42	1.974(1.21–4.39)	1.874. (1.178–3.360)*

*P<0.05

## Discussion

Pregnant women are one of the vulnerable groups of populations to develop nutritional anemia. Hence, WHO recommends a standard dose of 30-60mg iron + 400 μg folic acid supplementation to be taken daily starting as early as possible. However, maternal adherence to the regimen plays a major role in the prevention and treatment of iron deficiency anemia. Thus, the aim of this study was to determine anemia prevalence, the rate of adherence to IFAS and associated factors among pregnant women.

Even though the CBC result was obtained only for 58% of the study participants the prevalence of anemia was 4.8%. This result is lower than other previous similar studies which ranged from 16% to 46.3% [1, 8, 10, 25, 26]. This might be due to improved prenatal care and better living standards since this study was conducted in urban setting (capital city of Ethiopia). Nutritional variation, increased awareness of pregnant women through health education and access to social media regarding IFAS and anemia might be other possible justifications.

This study revealed that 159 (63.6%) of the participants were adherent to IFAS which is concordant with the study conducted in south India 64.7% [27] and Eritrean refugee camps, in northern Ethiopia 64.7% [4]. But it was higher than the study conducted in Mecha district, Western Amhara 20.4%, North Western Zone of Tigray 37.2%, Debre Tabor 44% and Northwest Ethiopia 55.3% [3, 19, 20, 28]. This inconsistency might be attributed to accessibility to quality health services as well as availability of IFAS for free since the study was conducted in tertiary care referral hospital. Furthermore, this study was conducted in urban area that might enhance awareness about IFAS and access to better information through different social media.

This study showed that only educational level and gestational age at the starting of ANC follow up had showed a significant association with adherence to IFAS during pregnancy. In the present study, adherence was higher by 1.87 times for women who started their ANC follow-up at first trimester as compared to those who started at second or third trimester. Similar findings were reported in different studies [3, 28]. The possible justification might be pregnant women who started early for ANC service might have more exposure to the health care provider and could acquire a better knowledge of perceived risk and benefit of IFAS to prevent anemia during pregnancy.

Pregnant women with educational status of secondary school completed and college level were at least 4times more likely to be adherent to IFAS than those who cannot read and write. This finding was supported by a study conducted in Mecha district, Western Amhara [19]. This is the fact that education would increase the women’s access to information through reading and understanding the benefit of the IFAS and possible consequences of anemia during pregnancy. However, parity, current anemia, and knowledge about IFAS were not significantly associated with adherence to IFAS during pregnancy in this study.

This study revealed that optimal health care providers’ counseling was the major reason for good adherence to IFAS. Similar reasons were reported in a study conducted in Mecha district, Western Amhara [19] and Northwest Ethiopia [3]. However, forgetfulness and fear of side effect were the leading reasons for poor adherence to IFAS. The finding of this study is supported by studies conducted in northwest Ethiopia [3] and Burji District, SNNPR of Ethiopia [24]. This problem could be addressed through effective counseling during ANC visit. Suggesting possible strategies to remember their tablets like placing the tablets in a spot that could be seen every day, alarming, correlating with natural occurrences like sunrise or sunset, lunch or dinner, toilet use or hand washing. Taking the tablets with meal to minimize the adverse effects might also improve adherence to IFAS.

This study showed that majority (78.2%) of the participants had good knowledge about IFAS which is higher than the study-conducted in Burji District, SNNPR of Ethiopia 46.7% [24]. The difference might be due to study setting difference. Thus, the present study was conducted at a tertiary referral hospital which is located in the capital city, having specialist health care providers and optimal client counseling compared to the study conducted in Burji District.

## Limitation of the study

Adherence to IFAS was assessed by asking the participants retrospectively about their IFAS taking behavior for the previous months that might have recall bias and might overestimate the adherence rate. Hemoglobin level was the only parameter used to determine anemia status and complete blood count was not available for some participants. Not all potential barriers to IFAS adherence had been investigated and the measurement of knowledge of respondents about anemia and IFAS is somehow subjective. Despite the limitations, the study tried to identify the most important factors for poor adherence and it reports new findings from a tertiary care setting in which several life-threatening obstetrical conditions are managed and anemia may be one of the common challenges.

## Conclusion

The overall prevalence of anemia in this study was only 4.8%, which is lower than most of previous studies. The rate of adherences was 63.6% and reasons for poor adherence were forgetfulness and fear of side effects. Educational level of the participants and first ANC follow-up starting time had a statistically significant association with adherences to IFAS. Therefore, educating pregnant women about anemia, encourage starting ANC follow-up as early as possible, providing adequate counseling regarding IFAS including taking with meal to minimize the side effects are recommended.

## Supporting information

S1 File Data collection tool. (DOCX)

S1 Raw data (SAV)

## Abbreviations

ANC: Antenatal care
Hb: Hemoglobin
IFAS: iron and folic acid supplementation
TASH: Tikur Anbessa Specialized Hospital
WHO: World Health Organization
