# Supplementary material for: Adherence to iron and folic acid supplementation and prevalence of anemia among pregnant women attending antenatal care clinic at Tikur Anbessa Specialized Hospital, Ethiopia
Source: PLoS One. 2020 May 4;15(5):e0232625. doi: 10.1371/journal.pone.0232625 (PMC7197778; doi:10.1371/journal.pone.0232625)
Supplement: S1 File — (DOCX) [file pone.0232625.s001.docx]

# Data collection tool

**Part I socio-demographic data**

1. Age ___________________

2. Maternal status: Married Unmarried Divorced Widowed

3. Religion: Orthodox Christian Muslim Protestant Catholic

If other please specify._____________________________

4. Educational level _________________________________

5. Occupational status: Housewife Government employed Self-employed

If other please specify.______________________

**Part II: Obstetrics related data**

1. Do you have a child (children)? Yes No

2. If yes how many numbers of child ___________________________

3. Gestational age at the time of first ANC visit___________________

4. Current gestational age____________________________________

**Part III: Knowledge of Anemia:**

1. Have you ever heard of anemia? Yes No

2. Pregnancy can cause anaemia? Yes No

3. Anaemia during pregnancy can is harmful to the foetus? Yes No

4. Which one of the following is/are the symptoms of anemia?

Palmer Pallor Tiredness Blurring of vision

Vertigo I do not know

If other please specify.______________________

5. Which one of the following will prevent anemia?

Taking Iron containing food Taking iron and folic acid supplements

Prevention of disease I don’t know

If other please specify.______________________

**Part IV: Adherence to Iron and folic acid supplementation**

1. During the previous one month, on average how many Iron and folic acid supplementation

tablets do you take? ___________

2. During the previous one week, how many Iron and folic acid supplementation

tablets do you take?___________________

3. What are the reasons for taking the Iron and folic acid supplementation?

Adequate explanation (medical advice) about the tablets by health care providers

Knowing it to prevent anemia

Getting the tablets for free

If other please specify.______________________

4. What are the reasons for not taking IFA tablets?

Fear of Side effects

Forgetfulness

Too many pills

Unpleasant tests

Fear that babies will become bigger

If other please specify.______________________

**Part V: Knowledge of Iron and folic acid supplementation**

1. Taking iron and folic acid tablet during pregnancy is important for the mother

Yes No

2. Taking iron and folic acid tablet during pregnancy is important for the infant

Yes No

3. Taking iron and folic acid tablets during pregnancy helps to prevent anemia

Yes No

4. Taking iron and folic acid tablets during pregnancy leads to too big baby

Yes No

5. Taking iron and folic acid tablets during pregnancy may helps to prevent birth defects.

Yes No

**Data collected from ANC follow up chart**

Complete blood count reports

RBC______________

HGB______________

HCT_______________

MCV_______________

**ቅጽ 1: የጥናቱ መረጃ ቅጽ (Amharic version(local language))**

**ክፍል 1፡**- **የጥናቱ ተሳታፊዎችን ማህበራዊ ጉዳዮች የሚመለከቱ ጥያቄዎች**

**1. እድሜ በአመት___________________**

**2**. **የጋብቻ ሁኔታ** ያገባች ያላገባች የፈታች የሞተባት

**3**. **ሃይማኖት**  ኦርቶዶክስ ሙስሊም ፕሮቴስታንት ካቶሊክ ሌላካለይግለጡ. _____________

**4.** **የትምህርት ደረጃ** መጻፍና ማንበብ የማትችል መጻፍና ማንበብ የምትችል

የመጀመሪያ ደረጃ 2ኛ ደረጃ እና ከዚያ በላይ

5. **የስራሁኔታ** የቤት-እመቤት የመንግስትሠራተኛ የግልሰራተኛ ሌላካለይግለጹ____________

**ክፍል 2፡ ከእርግዝና ጋር ተያያዥነት ያላቸዉ ጥያቄዎች**

1. ልጅ(ጆች) አሉዎት? አዎ የለኝም፡

**2.** አዎ ከሆነ መልስዎት ስንት ልጅ(ጆች) አሉዎት?_________________________

3. በዚህ የእርግዝና ወቅት ለመጀመሪያ ጊዜ ለእርግዝና ክትትል ጤና ተቋም የመጡት የስንት ሳምንት እርጉዝ እያሉ ነዉ?________________________________________

4. በአሁኑ ሰአት ስንትኛ የእርግዝና ሳምንት ላይ ነዎት?_______________________

**ክፍል 3፡ የደም ማነስ እዉቀትን የተመለከቱ ጥያቄዎች**

**1. ደም ማነስ ሲባል ሰምተዉ ያዉቃሉ?** አዎ የለም

**2. የደም ማነስ** በእርግዝና ምክንያት ሊመጣ ይችላል? አዎ አይችልም

**3. በእርግዝና ወቅት የሚከሰት ደም ማነስ ጽንሱን ሊጎዳ ይችላል?**  አዎ አይችልም

4 **የደም ማነስ ምልክት የቱ ነዉ/ናቸዉ?**

የእጅ መዳፍ መገርጣት(መንጣት) የድካም ስሜት ብዥታ አላዉቀዉም ሌላ___________________________

5. የደም ማነስ መከላከያ መንገድ የቱ ነዉ/ናቸዉ?

በአይረን ንጥረነገር የበለፀጉ ምግቦችን መመገብ

አይረን እና ፎሊክ አሲድ ቅይጥ ታብሌት መዉሰድ

በሽታን መከላከል አላዉቀዉም

ሌላ___________________________

**ክፍል 4፡ አይረነ እና ፎሊክ አሲድ ታብሌት አወሳሰድ እና ተያያዥ የጤና ሁኔታን የሚያስሱ** **ጥያቄዎች**

1. ባለፈዉ አንድ ወር ዉስጥ በአማካኝ ስንት አይረን እና ፎሊክ አሲድ ታብሌቶችን ወስደዋል? __________________________________

2. ባለፈዉ አንድ ሳምንት ዉስጥ ስንት አይረን እና ፎሊክ አሲድ ታብሌቶችን ወስደዋል? _______________________________

3. አይረን እና ፎሊክ አሲድ ታብሌት የማይወስዱበ ምክንያት ምንድን ነዉ?

የጎንዮሽ ጉዳት በመፍራት ስለምረሳዉ ደስ የማይል ጣዕም ስላላው ታብሌቱ ብዛት ስላለዉ ጥቅሙን ስለማላውቀው

ግዙፍ ልጅ እወልዳለሁ ብየ ስለምፈራ

ሌላ___________________________

**ክፍል 5፡ አይረን እና ፎሊክ አሲድ ታብሌት እዉቀትን የተመለከቱ ጥያቄዎች**

1. አይረን እና ፎሊክ አሲድ ታብሌት ሲባል ሰምተዉ ያዉቃሉ? አዎ አላውቅም

2. አይረን እና ፎሊክ አሲድ ታብሌት በእርግዝና ጊዜ መዉሰድ ለእናትዮዋ ጠቃሚ ነዉ

አዎ አይ

3. አይረን እና ፎሊክ አሲድ ታብሌት በእርግዝና ጊዜ መዉሰድ ለተረገዘዉ ልጅ ጠቃሚ ነዉ

አዎ አይ

4. አይረን እና ፎሊክ አሲድ ታብሌት በእርግዝና ወቅት መዉሰድ የደም ማነስን ይከላከላል

አዎ አይ

5. አይረን እና ፎሊክ አሲድ ታብሌት በእርግዝና ወቅት መዉሰድ የተረገዘዉን ልጅ ግዙፍ

እንዲሆን ያደርግዋል አዎ አይ

6. አይረን እና ፎሊክ አሲድ ታብሌት በእርግዝና ወቅት መዉሰድ የተረገዘዉን ልጅ ማህፀን

ዉስጥ እያለ የሚፈጠር የአካል ጉዳትን ለመከላከል ያግዛል

1. አዎ 2.አይ
